# Histological improvement of fibrosis in patients with hepatitis C who achieved a 5-year sustained virological response to treatment with direct-acting antivirals

**DOI:** 10.1007/s00535-024-02165-0

**Published:** 2024-11-25

**Authors:** Takayuki Iwamoto, Yasutoshi Nozaki, Takanori Inoue, Takahiro Suda, Rui Mizumoto, Yuki Arimoto, Takashi Ohta, Shinjiro Yamaguchi, Yoshiki Ito, Yoshiko Sudo, Michiko Yoshimura, Machiko Kai, Yoichi Sasaki, Yuki Tahata, Hayato Hikita, Tetsuo Takehara, Hideki Hagiwara

**Affiliations:** 1 Department of Gastroenterology and Hepatology, Kansai Rosai Hospital, 3-1-69, Inabaso, Amagasaki, Hyogo 660-8511 Japan; 2 Department of Diagnostic Pathology, Kansai Rosai Hospital, Hyogo, Japan; 3 Department of Gastroenterology and Hepatology, Osaka University Graduate School of Medicine, Osaka, Japan

**Keywords:** Liver fibrosis, Hepatitis C virus, Direct-acting antiviral, Sustained virological response

## Abstract

**Background:**

The histological improvement in liver fibrosis in patients with hepatitis C who achieved a sustained virological response (SVR) to direct-acting antiviral (DAA) treatment has not been comprehensively investigated. Therefore, we assessed the histological changes in liver fibrosis among patients with hepatitis C who underwent long-term follow-up after achieving SVR to treatment with DAA.

**Methods:**

This retrospective study enrolled 71 patients with hepatitis C who achieved SVR to treatment with DAA. Changes in histological liver fibrosis and fibrosis biomarkers (hyaluronic acid, type 4 collagen 7S, Mac-2 binding protein glycosylation isomer, autotaxin, and Fibrosis-4 index) were assessed before and 5 years after treatment. Transient elastography using the FibroScan® device was performed 5 years after treatment. Advanced fibrosis and cirrhosis were defined as Ishak fibrosis scores of ≥ 4 and ≥ 5, respectively.

**Results:**

Histological liver fibrosis significantly regressed after SVR. Fibrosis biomarkers were significantly reduced after SVR. Transient elastography was the most helpful after evaluating the predictive performance of advanced fibrosis and cirrhosis after SVR, with an area under the receiver operating characteristic curve of 0.965 and a cut-off value of 6.75 kPa. The cut-off values of serum fibrosis biomarkers for identifying advanced fibrosis and cirrhosis after SVR were lower than those before treatment.

**Conclusions:**

Long-term SVR to treatment with DAA ameliorated histological liver fibrosis. Noninvasive tests helped predict the degree of liver fibrosis after SVR, but their cut-off values should be redefined to avoid underestimation of liver fibrosis.

**Supplementary Information:**

The online version contains supplementary material available at 10.1007/s00535-024-02165-0.

## Introduction

Hepatitis C virus (HCV) infection, the global leading cause of cirrhosis and hepatocellular carcinoma (HCC), is a significant public health concern [1–3]. The introduction of interferon (IFN)-free direct-acting antiviral (DAA) regimens has significantly improved the outcomes of hepatitis C treatment [4–6]. Moreover, these regimens have expanded the range of potential candidates, including those with advanced age, a history of unsuccessful treatment, and decompensated cirrhosis, given their better tolerability and shorter treatment periods than IFN treatment [4]. Clinical trials and real-world cohort studies have shown that patients with hepatitis C who received IFN-free DAA regimens have sustained virological response (SVR) rates of approximately 100% [4–7].

HCV eradication offers several benefits, including decreased liver fibrosis and suppressed HCC development [8–13]. Furthermore, long-term histological improvement of liver fibrosis after anti-HCV treatment is reported in patients who achieved SVR to IFN treatment [8–10]. Studies using paired liver biopsies before and after treatment have shown fibrosis regression rates ranging from 38 to 82% in patients who achieved SVR to IFN treatment over prolonged intervals of approximately 5 years [8–10]. In contrast, advanced fibrosis after IFN treatment is a risk factor for HCC after SVR [14]. Therefore, patients with SVR with no decreasing or progressing fibrosis need to be identified and carefully monitored to reduce progression to HCC and liver-related death.

Liver biopsy remains the gold standard for evaluating liver fibrosis. However, recently, clinicians often avoid liver biopsies for liver fibrosis evaluation after SVR, mainly because of its invasiveness. Consequently, noninvasive tests (NITs), such as serum biomarkers and indices, ultrasound elastography, and magnetic resonance elastography, are becoming popular for liver fibrosis assessment. Moreover, increasing reports using NITs have shown post-treatment amelioration of liver fibrosis in patients who achieved SVR to DAA treatment [15–17]. However, limited studies have simultaneously evaluated NITs and histological assessments, and improvements in NIT measurements may reflect improved inflammatory activity. Notably, previous studies have evaluated histological liver fibrosis before and after treatment in patients who achieved SVR to DAA treatment. However, these studies had a short observation period after SVR and demonstrated inconsistent results [18–20]. Therefore, there is no evidence of liver fibrosis reduction over an extended follow-up period after DAA-induced SVR, warranting further investigation.

We evaluated the long-term histological changes in the degree of liver fibrosis before and after treatment with DAA in patients with SVR. Simultaneously, NITs were performed to investigate their ability to predict the stage of liver fibrosis.

## Methods

### Patients

Between December 2013 and July 2017, 409 patients were diagnosed with chronic hepatitis C and received treatment with DAA at our institution. Of these, 79 patients who achieved SVR to the treatment underwent paired pre- and post-treatment liver histological examinations.

The inclusion criteria were as follows: (1) complete antiviral treatment; (2) undetectable serum HCV at the end of treatment (EOT) and 24 weeks after the EOT (SVR24); and (3) paired pre- and post-treatment liver histological examination. All post-treatment histological examinations were performed 5 years after the EOT. None of the patients developed co-infection with hepatitis B or human immunodeficiency virus during the study period. The exclusion criteria were as follows: (1) other chronic liver diseases, such as autoimmune hepatitis, primary biliary cholangitis, drug-induced liver injury, or alcohol‐associated liver diseases (habitual alcohol intake > 60 g/day) before treatment, and (2) liver specimens unsuitable for histological evaluation with tissue sections < 10 mm or < 3 portal tracts. Two patients with other chronic comorbidities on pre-treatment histological evaluation, two with habitual alcohol intake > 60 g/day, and four with unsuitable liver specimens were excluded from the study. Finally, 71 patients with adequate liver specimens for histological evaluation pre- and post-treatment were enrolled. The patient flowchart is presented in Supplementary Figure S1.

Demographic information, including age, sex, HCV genotype, history of anti-HCV treatment, history of diabetes mellitus treatment, and alcohol intake, was retrospectively collected. The study was approved by the Institutional Review Board of Kansai Rosai Hospital (Approval No. 23E041g) and Osaka University Graduate School of Medicine (Approval No. K23182) and conducted following the Declaration of Helsinki. All patients provided written informed consent for liver biopsy pre- and post-treatment.

### Antiviral treatment

IFN-based DAA regimen was administered to 23 of the 71 patients analyzed. IFN-based DAA regimens included simeprevir 100 mg/day for 12 weeks plus pegylated interferon (PegIFN) alfa-2b and ribavirin for 24 weeks (*n* = 22) or vaniprevir 300 mg twice daily for 12 weeks plus PegIFN alfa-2b and ribavirin for 24 weeks (*n* = 1). PegIFN alfa-2b was administered at a 1.5 µg/kg dose once weekly. Based on body weight, ribavirin was administered at 600–1000 mg/day. The remaining 48 patients received IFN-free DAA regimens. Patients with HCV genotype 1 were treated with one of the following IFN-free DAA regimens: ledipasvir (90 mg) and sofosbuvir (400 mg) daily for 12 weeks (*n* = 34); ombitasvir (25 mg), paritaprevir (150 mg), and ritonavir (100 mg) daily for 12 weeks (*n* = 4); or elbasvir (50 mg) and grazoprevir (100 mg) daily for 12 weeks (*n* = 1). All patients with HCV genotype 2 treated with IFN-free regimens received sofosbuvir (400 mg) and weight-based ribavirin daily for 12 weeks (*n* = 9). SVR was defined as the absence of detectable HCV RNA at the EOT and 24 weeks after the EOT.

### Histological evaluation of liver specimens

Seventy of the 71 patients analyzed underwent histological evaluation using pre- and post-treatment liver biopsy specimens. The remaining patient underwent surgery for HCC before and after treatment, and the surgical specimens obtained at these time points were evaluated. Post-treatment specimens were collected from all patients 61 (interquartile range [IQR]: 60–61) months after the EOT. All liver biopsy specimens were obtained using a 16G BARD® MAX-CORE® Disposable Core Biopsy Instrument (Bard, Tempe, AZ, USA) under ultrasound guidance. Liver specimens were immediately fixed in 10% formalin buffered for approximately 24 h at room temperature, embedded in paraffin wax, cut into serial sections 2–3-µm thick, and stained with hematoxylin and eosin and Azan Mallory. This study’s histopathological findings were re-evaluated for research purposes. Specimens were coded, and two experienced pathologists blinded to clinical information evaluated inflammatory activity grading and fibrosis staging using a light microscope (Olympus, Shinjuku-ku, Tokyo, Japan). The field number of the microscope was standardized to 22 to assess focal lytic necrosis, apoptosis, and focal inflammation. Discordances between pathologists regarding the assessment of histological findings were resolved by reaching a consensus through discussion. Eligibility criteria for histological evaluation were as follows: specimens ≥ 10 mm with at least three portal tracts. Inflammatory activity grading and fibrosis staging were assessed using the modified histological activity index (mHAI) and Ishak fibrosis score [21]. Advanced fibrosis and cirrhosis were defined as Ishak fibrosis scores of ≥ 4 and ≥ 5. The proportion of hepatocytes affected by lipid droplets was quantitatively assessed, as described in the next section.

### Quantitative evaluation of hepatic steatosis

For quantitative image analysis of liver steatosis, 2–3-µm tissue sections were stained with hematoxylin and eosin to study lipid droplets. The slides were scanned into VSI files using SLIDEVIEW VS200 (Olympus, Shinjuku-ku, Tokyo, Japan), and the proportion of hepatocytes affected by lipid droplets was determined using HALO software (Indica Labs, Albuquerque, NM, USA). Hepatic steatosis was defined as the presence of > 5% of hepatocytes affected by lipid droplets. Samples were graded based on the nonalcoholic fatty liver disease activity score as follows: grade 1 (5–33% of hepatocytes affected), grade 2 (34–66% of hepatocytes affected), and grade 3 (> 66% of hepatocytes affected) [22].

### Noninvasive assessment of liver fibrosis

The following serum biomarkers and index were assessed as fibrosis biomarkers at treatment initiation and during post-treatment histological evaluation: hyaluronic acid (HA), type IV collagen 7S (4COL7S), Mac-2 binding protein glycosylation isomer (M2BPGi), autotaxin (ATX), and fibrosis-4 (FIB-4) index. Serum HA, 4COL7S, M2BPGi, and ATX levels were measured using a latex agglutination turbidimetric immunoassay (JCA-BM8000 series; JEOL Ltd., Akishima, Tokyo, Japan), chemiluminescent enzyme immunoassay (LUMIPULSE®L2400; Fujirebio Inc., Minato-ku, Tokyo, Japan), lectin-antibody sandwich immunoassay (HISCL-5000; Sysmex Corp., Kobe, Hyogo, Japan), and two-site immunoenzymometric assay (AIA-2000; TOSHO., Ohta-ku, Tokyo, Japan). The FIB-4 index was calculated using the following formula: FIB-4 = age (years) × aspartate aminotransferase (AST) (U/L) / platelet count (10^9^/L) × alanine aminotransferase (ALT) (U/L)^1/2^ [23]. Transient elastography using FibroScan® (Echosens, Paris, France) was performed only during post-treatment histological evaluation. An experienced physician or sonographer performed liver stiffness measurements (LSM) and controlled attenuation parameters. At least 10 measurements were performed to obtain a reliable median LSM with a success rate of ≥ 60% and an IQR/med of ≤ 0.2. The median time interval between FibroScan® and post-treatment histological evaluation was 38.5 (IQR: 5–60) days.

### Laboratory tests

We performed blood count (platelet count) and biochemical tests (albumin, AST, ALT, α-fetoprotein, and glycated hemoglobin A1c [HbA1c]) following standard procedures at baseline and 5 years after the EOT. Serum HCV-RNA levels were measured using the Roche Cobas 8800 system (Cobas® HCV; Roche Diagnostics K.K., Minato-ku, Tokyo, Japan). HCV genotype was determined by polymerase chain reaction using genotype-specific primers (BEX Co. Ltd., Itabashi-ku, Tokyo, Japan).

### Statistical analysis

Categorical variables are presented as numbers and percentages. Continuous variables are presented as median and IQR. Baseline characteristics between the two groups were compared using the Mann–Whitney U test for continuous variables and Fisher’s exact test for categorical variables. Differences between categorical and continuous variables in paired samples at baseline and 5 years after the EOT were analyzed using the Wilcoxon signed-rank test or McNemar test. Repeated measures ANOVA with Bonferroni corrections were used to compare continuous variables (serum HA and FIB-4 index) at baseline and 24 weeks after EOT and annually until 5 years after EOT. Trends in Ishak fibrosis score and NIT measurements were assessed using the Jonckheere-Terpstra test. The predictive performance of NITs for the liver fibrosis stage was evaluated using receiver operating characteristic (ROC) curve analysis, and areas under the ROC curves (AUROC) were calculated. AUROC values were compared using the DeLong test. The cut-off value for each NIT was the point at which the Youden index (sensitivity + specificity − 1) was maximized. The sensitivity, specificity, positive predictive value, negative predictive value, and accuracy were calculated to identify advanced liver fibrosis and cirrhosis. Statistical analyses were performed using SPSS 29.0 (SPSS Inc., Chicago, IL, USA). A P-value < 0.05 was considered statistically significant.

## Results

### Patient characteristics and noninvasive test results at baseline and during long-term follow-up after SVR

Clinical characteristics at baseline and 5 years after the EOT are summarized in Table 1. The number of patients with a treatment history of diabetes mellitus increased significantly (*P* = 0.02). We could not present HbA1c at 5 years after treatment because of the missing values. HCV RNA was undetected in all patients after treatment. Five years after EOT, the serum AST, ALT, and alpha-fetoprotein levels decreased significantly (*P* < 0.01); however, albumin levels and platelet counts increased significantly (*P* < 0.01). Levels of all fibrosis biomarkers decreased significantly 5 years after the EOT. Temporal changes in serum HA and FIB-4 index from baseline to 5 years after EOT are presented in Supplementary Figure S2. The serum HA and FIB-4 index decreased significantly 1 year after EOT, after which they remained flat (Supplementary Table S1).

**Table 1:** Clinical characteristics of the 71 patients with paired histologic evaluations

Factor	Baseline	Five years after the EOT	*P* value
Age, years (IQR)	65 (59–71)	70 (64–76)	< 0.01
Sex (male/female)	23/48		
Body mass index, kg/m^2^ (IQR)	22.5 (21.4–25.3)	23.0 (20.4–25.0)	0.94
Habitual alcohol intake 20–60 g/day, n (%)	5 (7.0)	5 (7.0)	1.00
History of diabetes mellitus treatment, n (%)	12 (16.9)	19 (26.8)	0.02
Treatment-naïve of HCV, n (%)	45 (63.4)		
Past HCC treatment, n (%)	1 (1.4)		
HbA1c, % (IQR)	5.7 (5.4–6.0)		
HCV genotype (1/2)	62/9		
HCV RNA, log_10_ IU/mL (IQR)	6.3 (5.5–6.7)	Negative	
Aspartate aminotransferase, U/L (IQR)	40 (28–66)	22 (19–24)	< 0.01
Alanine aminotransferase, U/L (IQR)	39 (26–73)	16 (12–22)	< 0.01
Albumin, g/dL (IQR)	4.2 (3.9–4.3)	4.3 (4.1–4.4)	< 0.01
Platelet count, × 10^3^/μL (IQR)	152 (110–182)	176 (149–210)	< 0.01
α-fetoprotein, ng/mL (IQR)	6.0 (5.0–12.0)	3.0 (2.0–4.0)	< 0.01
Hyaluronic acid, ng/mL (IQR)	108.0 (42.0–216.0)	48.0 (27.0–76.0)	< 0.01
Type4 collagen 7S, ng/mL (IQR)	5.4 (4.3–7.1)	2.9 (2.4–3.7)	< 0.01
M2BPGi, unit (IQR)	1.7 (1.1–3.0)	0.9 (0.7–1.2)	< 0.01
Autotaxin, mg/mL (IQR)	1.5 (1.1–2.0)	1.1 (0.9–1.4)	< 0.01
FIB-4 index (IQR)	3.06 (1.98–4.21)	1.99 (1.63–2.41)	< 0.01
Liver stiffness measurement, kPa (IQR)		5.1 (3.8–6.7)	
Controlled attenuation parameter, dB/m (IQR)		232 (199–267)	

FIB-4: Fibrosis 4. HbA1c: glycated hemoglobin A1c. HCC: hepatocellular carcinoma. HCV RNA: hepatitis C virus ribonucleic acid. IQR: interquartile range. M2BPGi: Mac-2 binding protein glycosylation isomer

Supplementary Tables S2 and S3 show the comparison of patient characteristics at baseline according to treatment regimen and advanced fibrosis, respectively. The number of patients with HCV genotype 2 in the IFN-based and IFN-free DAA groups differed significantly. At baseline, patients with advanced fibrosis had significantly higher serum AST, ALT, and alpha-fetoprotein levels, and all fibrosis biomarkers and significantly lower serum albumin levels and platelet counts than those without advanced fibrosis. However, there was no significant difference between the two groups in habitual alcohol intake and metabolic factors including body mass index (BMI), history of diabetes mellitus treatment, and HbA1c.

### Histological evaluation at baseline and long-term follow-up after SVR

**Fig. 1:**
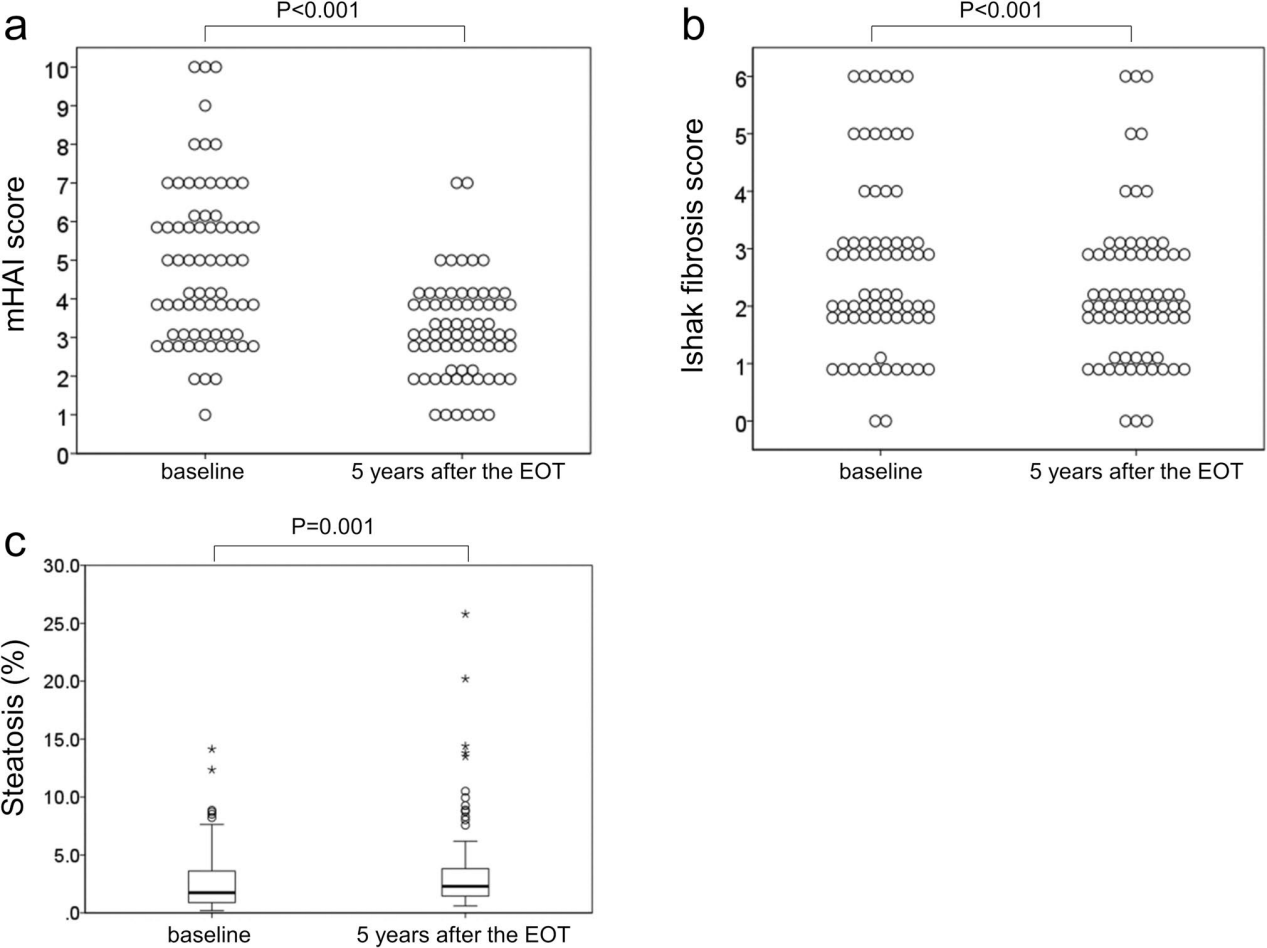
Changes in histological inflammation, fibrosis, and steatosis at baseline and 5 years after the end of treatment. **a** Change in modified histological activity index score. **b** Change in Ishak fibrosis score. **c** Change in the proportion of hepatocytes affected by lipid droplets estimated using quantitative assessment. The horizontal line through each box represents the median, and each box represents data from the 25th to the 75th percentile. The separate asterisks and circles represent outliers

Figure 1 presents the results of the histological assessment of inflammation, fibrosis, and steatosis at baseline and 5 years after the EOT.

At baseline histological evaluation, the total mHAI scores ranged from 1 to 10 (out of 18). Among the included patients, four (5.6%) had moderate chronic hepatitis (mHAI scores, 9–12), 46 (64.8%) had mild chronic hepatitis (mHAI scores, 4–8), and 21 (29.6%) had minimal chronic hepatitis (mHAI scores, 0–3). Ishak fibrosis scores ranged from 0 to 6. Moreover, 12 patients (16.9%) had cirrhosis (Ishak fibrosis score, 5–6), 22 (31.0%) had bridging fibrosis (Ishak fibrosis score, 3–4), 35 (49.3%) had portal fibrosis (Ishak fibrosis score, 1–2), and two (2.8%) had no detectable fibrosis. Quantitative assessment of hepatic steatosis (≥ 5% of hepatocytes affected) revealed grade 1 steatosis in 12 patients (16.9%), three of whom also had the hepatocellular ballooning characteristic of steatohepatitis.

Histological evaluation performed 5 years after the EOT revealed total mHAI scores ranging from 1 to 7. Twenty-six patients (36.6%) had mild chronic hepatitis, and 45 (63.4%) had minimal chronic hepatitis. The Ishak fibrosis scores ranged from 0 to 6. Notably, 5 patients (7.0%) had cirrhosis, 19 (26.8%) had bridging fibrosis, 44 (62.0%) had portal fibrosis, and three (4.2%) had no detectable fibrosis. Quantitative assessment of hepatic steatosis revealed grade 1 steatosis in 16 patients (22.5%), four of whom also exhibited the hepatocellular ballooning characteristic of steatohepatitis.

### Histological changes at baseline and long-term follow-up after SVR

**Fig. 2:**
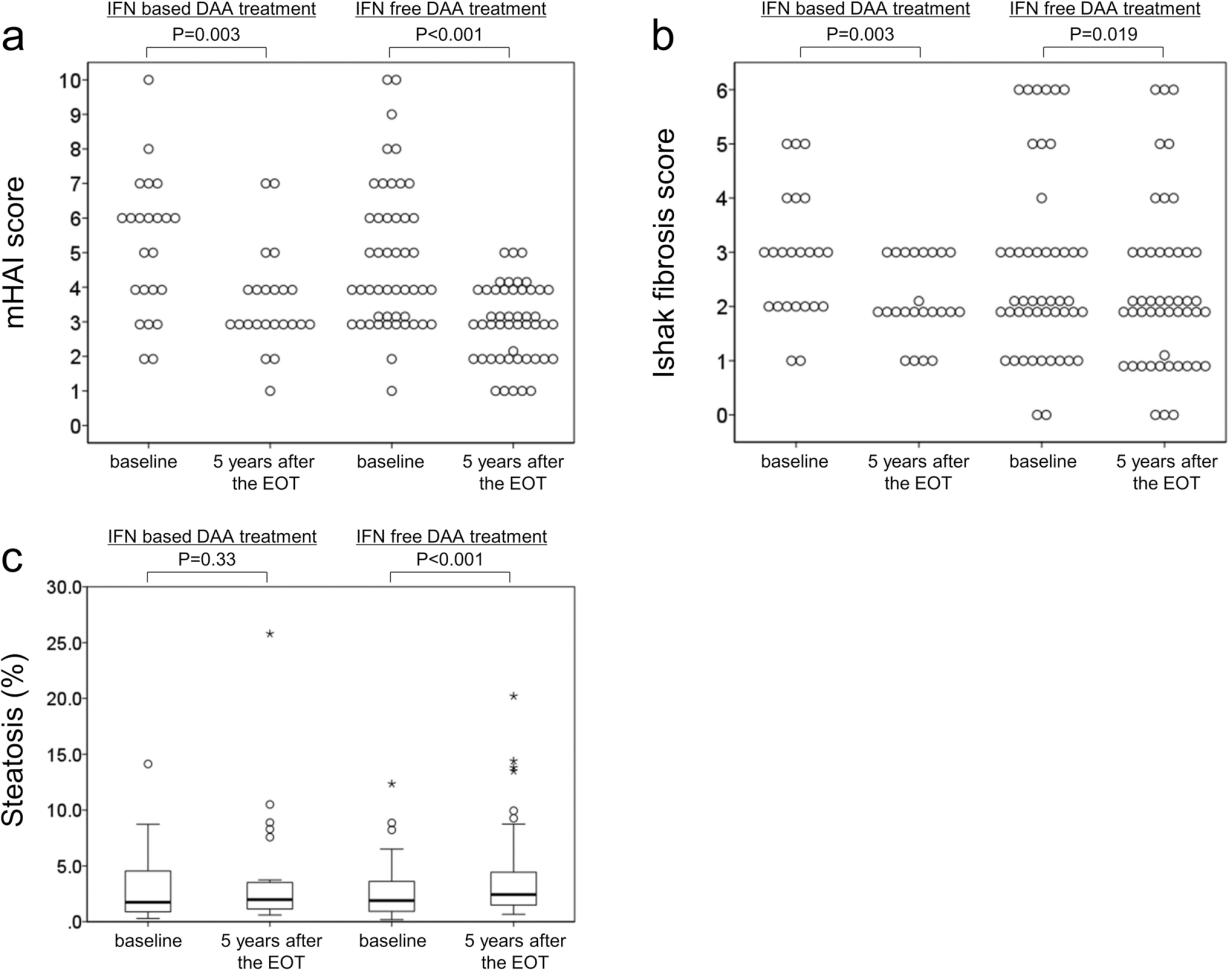
Changes in histological inflammation, fibrosis, and steatosis at baseline and 5 years after the end of treatment in interferon (IFN)-based (*n* = 23) and IFN-free direct-acting antiviral (DAA) treatment groups (*n* = 48). **a** Change in modified histological activity index score. **b** Change in Ishak fibrosis score. **c** Change in the proportion of hepatocytes affected by lipid droplets estimated using quantitative assessment. The horizontal line through each box represents the median, and each box represents data from the 25th to the 75th percentile. The separate asterisks and circles represent outliers

The mHAI score had significantly decreased (*P* < 0.001; Fig. 1a). Overall, 38 patients (53.5%) showed a ≥ 2-point improvement in the mHAI score, whereas 11 (15.5%) exhibited a ≥ 1-point worsening. The median mHAI score decreased from 5 (IQR: 3–6) at baseline to 3 (IQR: 2–4) at 5 years after EOT. The Ishak fibrosis score also significantly decreased (*P* < 0.001; Fig. 1b). Notably, 32 patients (45.1%) showed a ≥ 1-point improvement in the Ishak fibrosis score, whereas 12 (16.9%) exhibited a 1-point worsening of the same. No patient demonstrated a > 2-point worsening in the Ishak fibrosis score. The median Ishak fibrosis score was unchanged, with 2 (IQR: 2–3) at baseline and 2 (IQR: 1–3) at 5 years after EOT. The Ishak fibrosis score significantly decreased in both groups of patients with and without advanced fibrosis at baseline (*P* = 0.002 and *P* = 0.04, respectively; Supplementary Figure S3). A significant decrease in fibrosis was also observed in patients with and without cirrhosis at baseline (*P* = 0.007 and *P* = 0.016, respectively; Supplementary Figure S4). Additionally, the mHAI and Ishak fibrosis scores in the IFN-based and IFN-free DAA treatment groups improved significantly 5 years after the EOT (mHAI, P = 0.003 and P < 0.001, respectively; Ishak fibrosis score, *P* = 0.003 and *P* = 0.019, respectively; Fig. 2a and b).

The proportion of hepatocytes affected by lipid droplets was 1.74% (IQR: 0.89–3.61) at baseline and significantly increased to 2.29% (IQR: 1.45–3.82) at 5 years after EOT (*P* < 0.01; Fig. 1c). Among the 12 patients diagnosed with hepatic steatosis at baseline, 6 had no hepatic steatosis on post-treatment histological evaluation. Conversely, among the 59 patients without hepatic steatosis at baseline, 10 were newly diagnosed with hepatic steatosis on post-treatment histological evaluation. The proportion of hepatocytes affected by lipid droplets in the IFN-based and IFN-free DAA treatment groups increased from 1.74% (IQR: 0.88–4.54) and 1.89% (IQR: 0.94–3.53) to 1.97% (IQR: 1.14–3.51) and 2.43% (IQR: 1.50–4.16), respectively. Additionally, the proportion of hepatocytes affected by lipid droplets in the IFN-free group significantly differed between baseline and 5 years after EOT (*P* < 0.001; Fig. 2c).

### Correlations between noninvasive test results and histological fibrosis stage

**Fig. 3:**
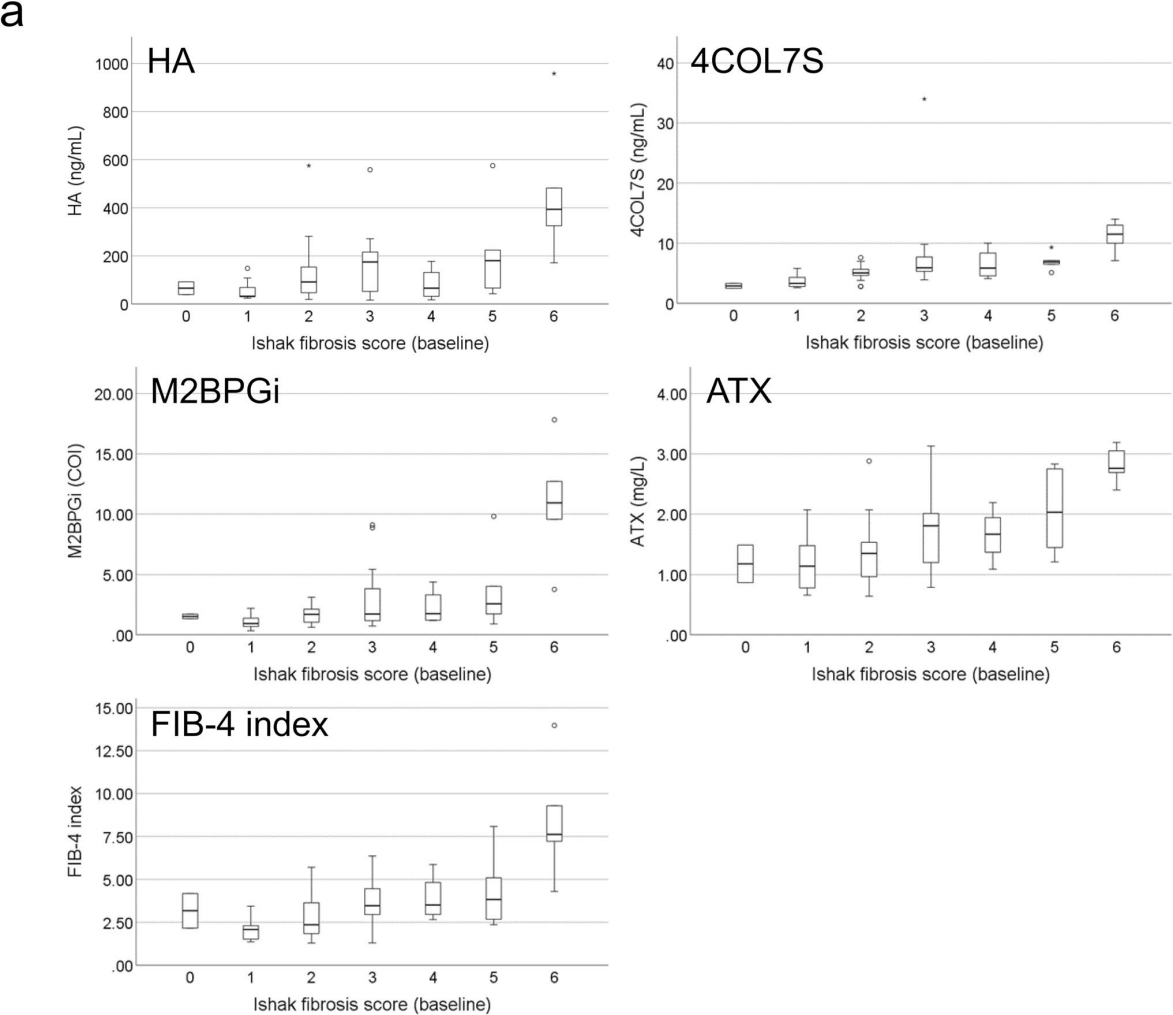

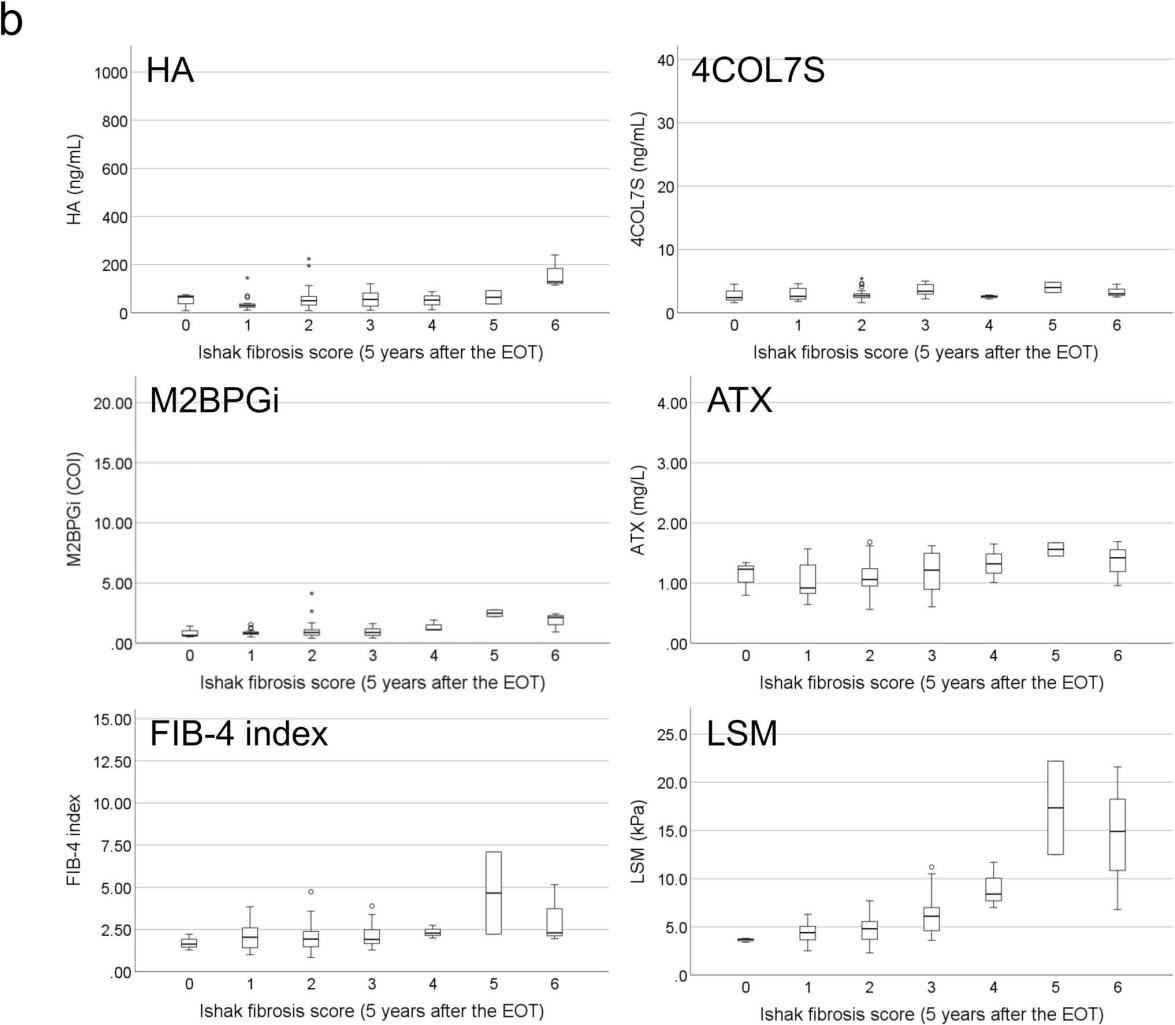
Correlations between Ishak fibrosis score and noninvasive fibrosis tests. **a** Correlation between Ishak fibrosis score and fibrosis biomarkers, including hyaluronic acid (HA), type IV collagen 7S (4COL7S), Mac-2 binding protein glycosylation isomer (M2BPGi), autotaxin (ATX), and FIB-4 index at baseline. **b** Correlation between Ishak fibrosis score and noninvasive tests, including HA, 4COL7S, M2BPGi, ATX, FIB-4 index, and liver stiffness measurements 5 years after the end of treatment. The horizontal line through each box represents the median, and each box represents data from the 25th to the 75th percentile. The separate asterisks and circles represent outliers

Figure 3 presents the correlation between NITs and Ishak fibrosis score. At baseline, the median values of all fibrosis biomarkers increased with increasing Ishak fibrosis score (*P* < 0.01 for all fibrosis biomarkers; Fig. 3a). At 5 years after EOT, the median values of LSM and fibrosis biomarkers, except the FIB-4 index, increased with increasing Ishak fibrosis score (HA: *P* = 0.01, 4COL7S: *P* = 0.03, M2BPGi: *P* = 0.02, ATX: *P* = 0.02, FIB-4 index: *P* = 0.07, and LSM: *P* < 0.01; Fig. 3b).

### Predictive performance of noninvasive assessment of advanced fibrosis and cirrhosis

**Fig. 4:**
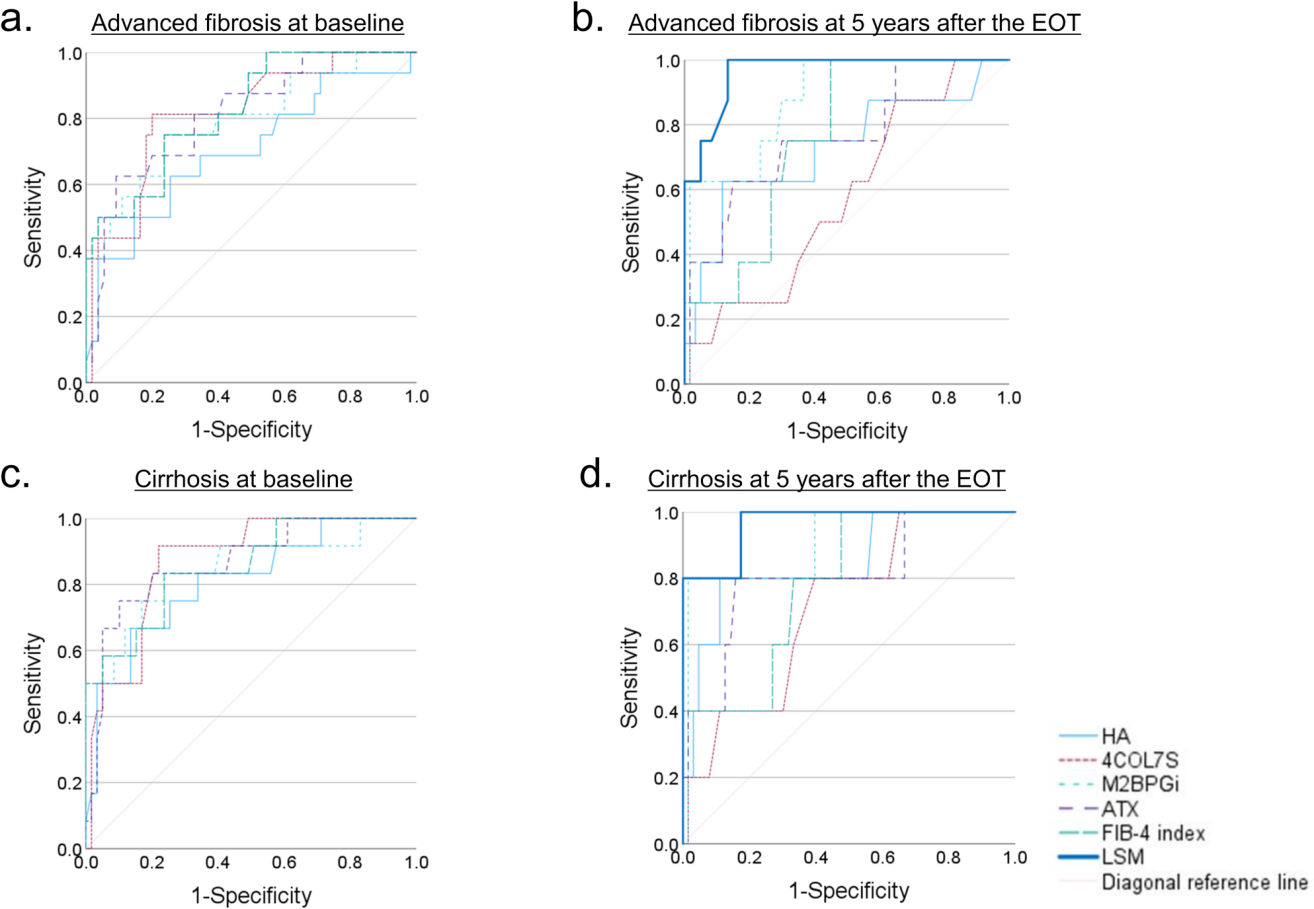
Predictive performance of noninvasive fibrosis test values for advanced fibrosis (Ishak fibrosis score ≥ 4) and cirrhosis (Ishak fibrosis score ≥ 5). **a** Areas under the receiver operating characteristic curves (AUROC) for diagnosing advanced fibrosis at baseline were 0.701, 0.815, 0.794, 0.813, and 0.823 for serum hyaluronic acid (HA), type IV collagen 7S (4COL7S), Mac-2 binding protein glycosylation isomer (M2BPGi), autotaxin (ATX) levels, and FIB-4 index, respectively. **b** AUROC for diagnosing advanced fibrosis 5 years after the end of treatment (EOT) were 0.714, 0.562, 0.864, 0.766, 0.751, and 0.964 for serum HA, 4COL7S, M2BPGi, ATX levels, FIB-4 index, and liver stiffness measurements (LSM), respectively. **c** AUROC for diagnosing cirrhosis at baseline are 0.809, 0.870, 0.847, 0.865, and 0.854 for serum HA, 4COL7S, M2BPGi, ATX levels, and FIB-4 index, respectively. **d** AUROC for diagnosing cirrhosis 5 years after the EOT were 0.836, 0.698, 0.897, 0.805, 0.777, and 0.965 for serum HA, 4COL7S, M2BPGi, ATX levels, FIB-4 index, and LSM, respectively

ROC analysis of each NIT in identifying pre-treatment advanced fibrosis (Ishak fibrosis score of 4–6) showed that all tests had an AUROC > 0.7, with 4COL7S, ATX, and FIB-4 index demonstrating particularly good predictive performance (AUROC > 0.8) (Fig. 4a and Table 2). There was no significant difference in predictive performance in identifying advanced fibrosis between each NIT, except between HA and FIB-4 index (Supplementary Table S4). ROC analysis of each NIT used in identifying advanced fibrosis 5 years after the EOT showed that all tests, except 4COL7S, had an AUROC > 0.7, with M2BPGi and LSM showing good predictive ability (AUROC > 0.8). Additionally, only LSM had an AUROC > 0.9, which was significantly higher than that of HA, 4COL7S, ATX, and FIB-4 index in identifying advanced fibrosis 5 years after EOT (Fig. 4b, Table 3 and Supplementary Table S5). Notably, the cut-off values of all fibrosis biomarkers for identifying advanced fibrosis decreased 5 years after the EOT. The cut-off value of LSM 5 years after the EOT was 6.75 kPa.

**Table 2:** Predictive performance of non-invasive tests for advanced fibrosis at baseline

Factor	AUROC	Cut-off	Sensitivity	Specificity	PPV	NPV	Accuracy
Hyaluronic acid	0.701	167.5	0.625	0.745	0.417	0.872	0.718
Type4 collagen 7S	0.815	6.40	0.813	0.800	0.542	0.936	0.803
M2BPGi	0.794	2.24	0.750	0.764	0.480	0.913	0.761
Autotaxin	0.813	2.12	0.625	0.909	0.667	0.893	0.845
FIB-4 index	0.823	3.76	0.750	0.764	0.480	0.913	0.761

AUROC: Area Under the Receiver Operating Characteristic. FIB-4: Fibrosis 4. M2BPGi: Mac-2 binding protein glycosylation isomer. NPV: negative predictive value. PPV: positive predictive value

**Table 3:** Predictive performance of non-invasive tests for advanced fibrosis five years after the EOT

Factor	AUROC	Cut-off	Sensitivity	Specificity	PPV	NPV	Accuracy
Hyaluronic acid	0.714	87.0	0.625	0.857	0.357	0.947	0.831
Type4 collagen 7S	0.562	2.45	0.875	0.333	0.143	0.955	0.394
M2BPGi	0.864	0.91	1.000	0.619	0.250	1.000	0.662
Autotaxin	0.766	1.42	0.625	0.841	0.333	0.946	0.817
FIB-4 index	0.751	1.95	1.000	0.540	0.216	1.000	0.592
LSM	0.964	6.75	1.000	0.867	0.500	1.000	0.882

AUROC: Area Under the Receiver Operating Characteristic. EOT: end of treatment. FIB-4: Fibrosis 4

LSM: liver stiffness measurement. M2BPGi: Mac-2 binding protein glycosylation isomer

NPV: negative predictive value. PPV: positive predictive value

ROC analysis of the ability of each NIT to identify pre-treatment cirrhosis (Ishak fibrosis score of 5–6) showed that all tests had AUROC > 0.8 with no significant difference (Fig. 4c and Supplementary Tables S4 and S6). ROC analysis of the ability of each NIT in identifying cirrhosis 5 years after the EOT showed that all tests, except 4COL7S, had AUROC > 0.7, with HA, M2BPGi, ATX, and LSM showing good predictive ability (AUROC > 0.8). Additionally, only LSM had an AUROC > 0.9, which was significantly higher than that of 4COL7S in identifying cirrhosis 5 years after the EOT (Fig. 4d and Supplementary Tables S5 and S7). The cut-off values of all fibrosis biomarkers for identifying cirrhosis also decreased 5 years after the EOT. The cut-off value of LSM for identifying cirrhosis after 5 years of EOT was also 6.75 kPa.

## Discussion

Liver fibrosis is the most critical factor directly associated with patient prognosis. Advanced liver fibrosis is the leading risk factor of HCC and can lead to fatal conditions, including decompensated cirrhosis and liver failure. Liver fibrosis was previously considered irreversible; however, recent reports have revealed that fibrosis can be ameliorated by therapeutic intervention that addresses the cause of chronic liver disease [24–27].

This study is the first to demonstrate an improvement in histological fibrosis over a substantially long period (a median of 60 months from EOT) by comparing histological evaluation before and after treatment with DAA. Furthermore, improvement in histological fibrosis was observed in patients with and without advanced fibrosis at baseline. Three previous studies have evaluated changes in histological fibrosis after DAA treatment [18–20]. However, these studies included a small number of patients (9–40) and performed histological evaluation relatively quickly after SVR, with median durations of 6 months [18], 10 months [19], and 19.2 months [20]. Two of these studies showed no improvement in histological fibrosis after DAA treatment [19, 20]; however, the remaining one reported that 38% (15/40) of patients had improved their Ishak fibrosis score by > 1 point at a median 6-month histological evaluation after SVR compared to that before SVR [18]. Conversely, improvement in fibrosis after SVR to IFN treatment has been reported over long-term intervals of approximately 5 years [8–10]. Furthermore, Pockros et al. suggested that better histological fibrosis improvement may be associated with a longer period of viral suppression after IFN treatment [28]. Therefore, the lack of improvement in histological fibrosis after DAA treatment in previous reports may have been due to the short observation period. Our findings showed that viral elimination after treatment with DAA improved histological fibrosis at 5 years after EOT.

In the current study, we also assessed changes in histological fibrosis according to the treatment regimen. The IFN-free and IFN-based DAA groups exhibited significant improvement in histological fibrosis. The proportion of HCV genotypes was significantly different between the two groups; however, the improvement in histological fibrosis was significant in both groups, even in patients with HCV genotype 1 (IFN-based DAA group; *n* = 23, IFN-free DAA group *n* = 39; Supplementary Figure S5). These results suggest that the anti-inflammatory effects of DAA treatment associated with HCV elimination contribute to the improvement of fibrosis, even without the antifibrotic effects of IFN [29].

Moreover, the finding that 12 patients (16.9%) had worsening Ishak scores is clinically important. Supplementary Tables S8 and S9 show the characteristics at baseline and 5 years after SVR in patients with and without histologic fibrosis progression after achieving SVR. The BMI and number of patients with habitual alcohol intake were significantly higher in patients with histological fibrosis progression than in those without histological fibrosis progression at baseline and 5 years after SVR. Moreover, AST, ALT, AFP, HA, 4COL7S, M2BPGi, and the FIB-4 index were significantly lower in patients with histological fibrosis progression than those without at baseline. At the same time, these factors showed either no significant differences or comparable levels even if they had a significant difference 5 years after the EOT. These findings suggest that a high BMI and alcohol consumption may influence the progression of histological fibrosis after SVR. Therefore, careful follow-up is desirable for patients with such background characteristics. However, since the number of patients with these factors was small in this study, further analysis involving a large cohort is needed to substantiate these findings.

Another strength of our study is the simultaneous assessment of liver fibrosis using histological evaluation and NITs at baseline and 5 years after the EOT with antiviral treatment. The use of NITs for the evaluation of fibrosis after SVR has not been established. The EASL guidelines state that noninvasive methods are unreliable after SVR and should not be used to assess the fibrosis stage after treatment [30]. The problem with evaluating the fibrosis stage after SVR using NITs is that improved NIT measurements may reflect improved inflammation due to HCV elimination. Therefore, to address this issue, NITs and histological evaluation should be performed simultaneously to confirm the diagnostic ability of NITs in assessing the fibrosis stage before and after SVR. Consistent with previous reports [15–17], all fibrosis biomarkers in our study had significantly decreased levels after treatment with DAA. Except for 4COL7S, all NITs could predict advanced-stage liver fibrosis and cirrhosis after SVR, with AUROCs of > 0.7 and > 0.75, respectively. Notably, M2BPGi (AUROC > 0.85) and LSM (AUROCs > 0.95) were exceptionally useful in predicting liver fibrosis after SVR. Although LSM is useful for diagnosing fibrosis after SVR, it is important to determine whether LSM at 5 years after EOT reflects only fibrosis without inflammation or includes inflammation. LSM showed no tendency to increase with increasing mHAI score 5 years after the EOT (*P* = 0.23) (Supplementary Figure S6). Supplementary Table S10 presents the characteristics of patients with and without high LSM (≥ 6.75 kPa). There was no difference between the two groups in terms of alcohol intake and metabolic factors, such as fatty liver and diabetes mellitus. We excluded patients with other chronic liver diseases, such as autoimmune hepatitis, primary biliary cholangitis, and drug-induced liver injury. Considering these results, LSM at 5 years after SVR mainly reflects fibrosis due to hepatitis C.

Furthermore, our results showed a greater decrease in the cut-off values of all fibrosis biomarkers for advanced-stage fibrosis after SVR than before treatment. Similar results were reported in a previous study on IFN treatment. Tachi et al. reported that fibrosis indices, namely the AST-to-platelet ratio index (APRI), FIB-4 index, and Forns index, can predict liver fibrosis even after SVR-to-IFN treatment, with an AUROC of ≥ 0.8 [31]. Significant differences in the cut-off values of these indices, especially APRI, were observed between the biopsy performed before and after treatment [31]. In our study, LSM was measured only after 5 years of EOT, and we could not evaluate changes in cut-off values before and after SVR. However, the cut-off value of 6.75 kPa for identifying advanced fibrosis after 5 years of EOT is equivalent to that for mild fibrosis (METAVIR fibrosis stage F0-1) in previous reports [32]. Therefore, serum fibrosis markers and elastography are promising for evaluating the long-term severity of liver fibrosis after SVR to DAA treatment. Notably, the underestimation of fibrosis might be attributed to the use of the conventionally reported cut-off value of NITs. Further validation is required to avoid the underestimation of fibrosis using NITs, including the establishment of appropriate cut-off values for NITs in patients with HCV after achieving SVR for DAA treatment.

The long-term course of NIT measurements after SVR is another notable finding of this study. In our results, serum HA and FIB-4 index decreased rapidly in their measurements until 1 year after the EOT, after which they remained stable or gradually decreased. Notably, several studies examining the long-term course of NITs before and after antiviral treatment for HCV have also reported a rapid decline in NITs over a short period (approximately 1 year after SVR), followed by a stable or gradual decline [17, 33, 34]. Thus, we speculate that the short-term decrease in NITs after DAA treatment reflects a reduction in inflammation, and the subsequent sustained decrease in NITs may reflect the degree of improvement in histological fibrosis. However, it is impossible to determine when NIT measurements accurately reflect histological liver fibrosis improvement after DAA-induced SVR. Additionally, previous reports have been inconsistent in their conclusions regarding the short-term improvement in histological liver fibrosis after DAA-induced SVR [18–20]. Therefore, repeated liver biopsies and NIT measurements after DAA-induced SVR might be necessary to elucidate these issues; however, they lack feasibility because of the invasiveness of liver biopsies.

Hepatic steatosis is a histological feature of HCV infection, and HCV is associated with the development of hepatic steatosis. However, the frequency at which it develops varies with viral genotype. In patients with HCV genotype 3, the virus is directly involved in the development of the fatty liver, and the frequency of hepatic steatosis complications is high. Moreover, achieving viral elimination with IFN treatment decreases histological steatosis in patients with HCV genotype 3 [35, 36]. However, in patients with HCV genotype 1–2, the development of hepatic steatosis is mainly associated with host factors. Therefore, viral elimination does not improve hepatic steatosis. A histological study after IFN treatment showed no change in fatty liver in patients with HCV genotype 1, regardless of treatment response [35]. Conversely, previous studies that evaluated hepatic steatosis after SVR to DAA treatment using NITs have reported inconsistent results with contradictory conclusions [37–41]. In the current study, hepatic steatosis did not improve over the long term after treatment with DAA. This may be due to the selection bias of our cohort, which included only patients with HCV genotype 1/2, in whom hepatic steatosis development is mainly associated with host factors. Previous studies have reported that increased BMI, concomitant diabetes mellitus, and human patatin-like phospholipase domain-containing 3 gene polymorphism were risk factors for progressive steatosis after DAA-induced SVR [42].

Unfortunately, this study lacked data on changes in glycated hemoglobin levels after SVR and host genetic factors, and we were unable to assess factors contributing to advanced steatosis.

This study has some limitations. First, given the invasiveness of liver biopsy, few patients consented to post-treatment liver biopsy compared to those treated with DAA, likely introducing patient selection bias. Second, although some authors have reported that liver specimens are adequate if they are > 15 mm and contain six or more portal tracts [19], this study included 21 liver specimens with tissue sections ≥ 10 mm and 3–5 portal tracts at histological evaluation 5 years after SVR. The size of the tissue specimen, especially 5 years after SVR, was influenced by our institution’s practice of using a single puncture with a 16-gauge needle for liver biopsy examinations to minimize the bleeding risk. The evaluation of fibrosis and inflammation in this study using specimens with a tissue length > 10 mm and 3–5 portal tracts might be less comprehensive compared to that in previous studies using larger tissue samples. Third, only a small number of patients with cirrhosis (12 cases, 16.9%) were enrolled. Therefore, we might have failed to thoroughly investigate whether HCV elimination improved fibrosis in patients with pre-treatment cirrhosis. Finally, we did not perform pre-treatment elastography-based LSM. We could not evaluate the correlation between pre- and post-treatment changes in liver stiffness and histological fibrosis. A larger prospective study is needed to confirm our findings. However, it may be difficult to achieve given the disadvantages of liver biopsy including its invasive nature.

In conclusion, long-term follow-up after SVR to treatment with DAA showed improvement in histological liver fibrosis. NITs also helped predict the extent of liver fibrosis after SVR to treatment with DAA. However, their cut-off values need to be redefined to avoid underestimating the degree of liver fibrosis after SVR.

## Supplementary Information

Below is the link to the electronic supplementary material.

Supplementary file1 (DOCX 1236 KB)
