# Supplementary material for: Histological improvement of fibrosis in patients with hepatitis C who achieved a 5-year sustained virological response to treatment with direct-acting antivirals
Source: J Gastroenterol. 2024 Nov 25;60(2):197–209. doi: 10.1007/s00535-024-02165-0 (PMC11794422; doi:10.1007/s00535-024-02165-0)
Supplement: Supplementary file 1 — Supplementary file1 (DOCX 1236 KB) [file 535_2024_2165_MOESM1_ESM.docx]

***Supplementary Materials***

**Histological improvement of fibrosis in patients with hepatitis C who achieved 　　a 5-year sustained virological response to treatment with direct-acting antivirals**

***Table of Contents***

Supplementary Figure S1...…………………………...……....…….……………………3

Supplementary Figure S2...…………………………...……....…….……………………4

Supplementary Figure S3...…………………………...……....…….……………………5

Supplementary Figure S4...…………………………...……....…….……………………6

Supplementary Figure S5...…………………………...……....…….……………………7

Supplementary Figure S6...…………………………...……....…….……………………8

Supplementary Table S1...………………………………………………...……...………9

Supplementary Table S2...………………………………………………...….…...…….10

Supplementary Table S3...………………………………………………...….…...…….11

Supplementary Table S4...………………………………………………...….…...…….12

Supplementary Table S5...………………………………………………...….…...…….13

Supplementary Table S6...………………………………………………...….…...…….14

Supplementary Table S7...………………………………………………...….…...…….15

Supplementary Table S8...………………………………………………...….…...…….16

Supplementary Table S9...………………………………………………...….…...…….17

Supplementary Table S10...………………………………………………...….…...…...18


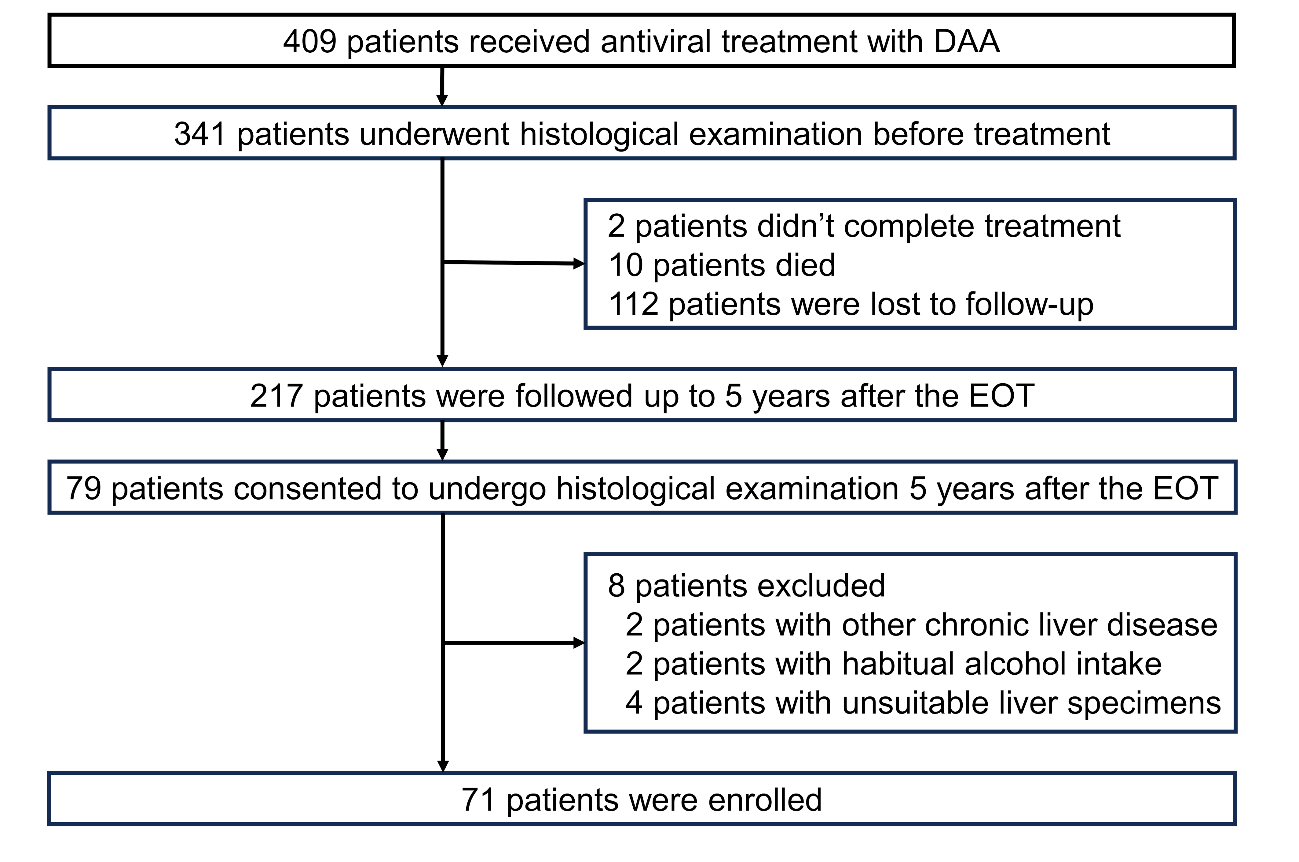


**Supplementary Figure S1.** Patient flowchart.

Between December 2013 and July 2017, 410 patients were diagnosed with chronic hepatitis C and received treatment with direct-acting antiviral at our institution, among whom 217 patients were followed up to 5 years after the end of treatment and 79 patients underwent paired pre- and post-treatment liver histologic examination. Eight patients met the exclusion criteria. Finally, 71 were enrolled in this study.


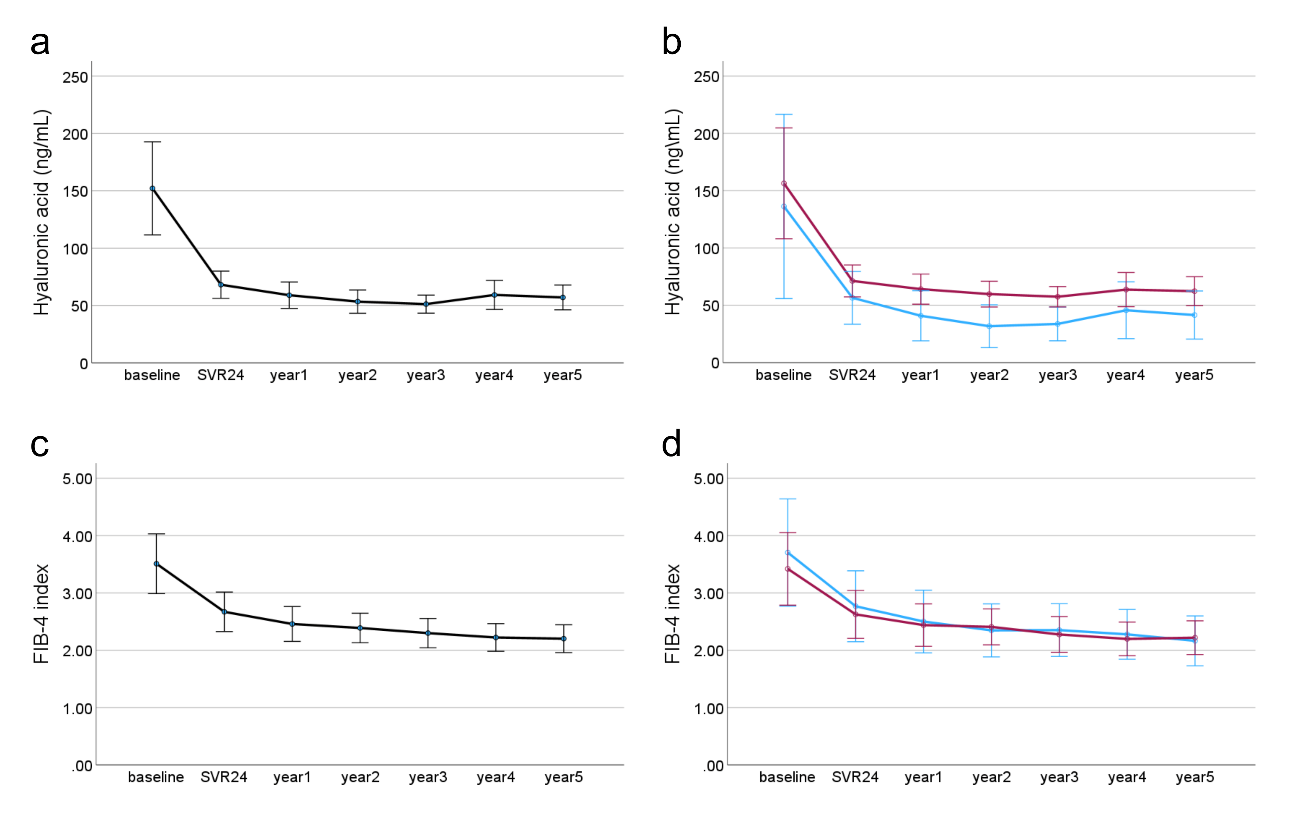


**Supplementary Figure S2.** Changes in hyaluronic acid and Fibrosis 4 (FIB-4) index among patients who achieved sustained virological response to treatment with direct-acting antiviral (DAA).

(a) Hyaluronic acid for all patients. (b) Hyaluronic acid based on treatment regimen. (c) FIB-4 index for all patients. (d) FIB-4 index based on treatment regimen.

Blue line, interferon (IFN)-based DAA regimens; red line, IFN-free DAA regimens.


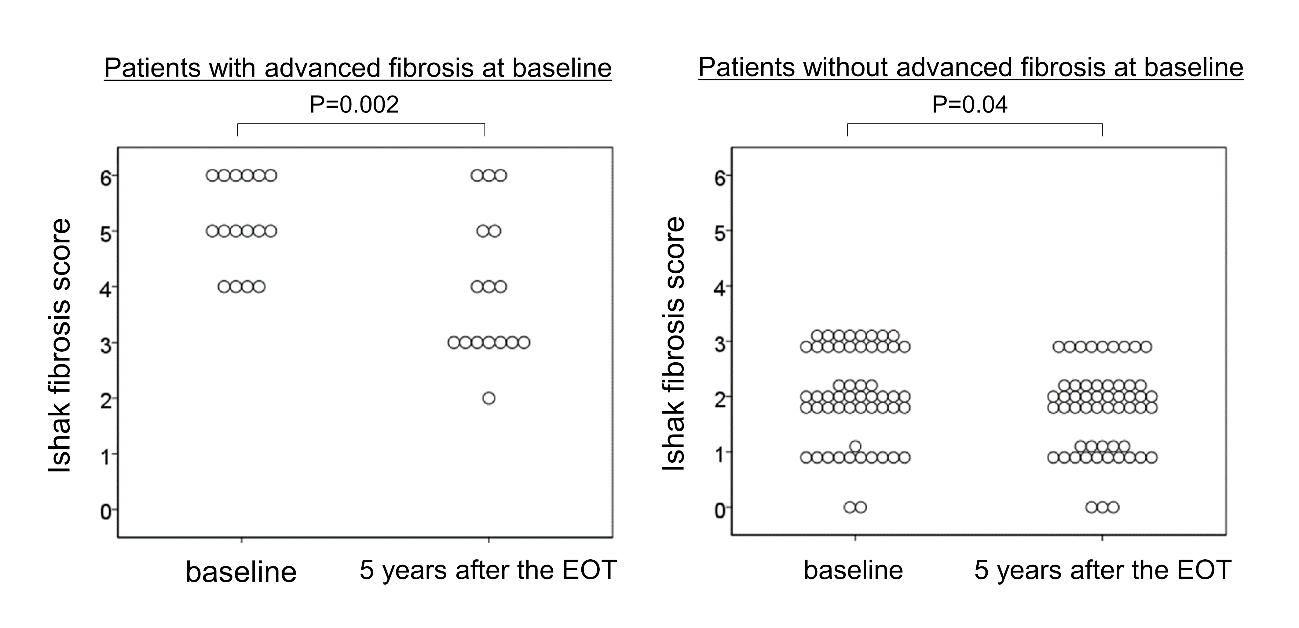


**Supplementary Figure S3.** Change in Ishak fibrosis score in patients with and without advanced fibrosis (Ishak fibrosis score ≥ 4) at baseline.

There were 16 and 55 patients with and without advanced fibrosis, respectively. The Ishak fibrosis score decreased significantly in patients with and without advanced fibrosis.

**
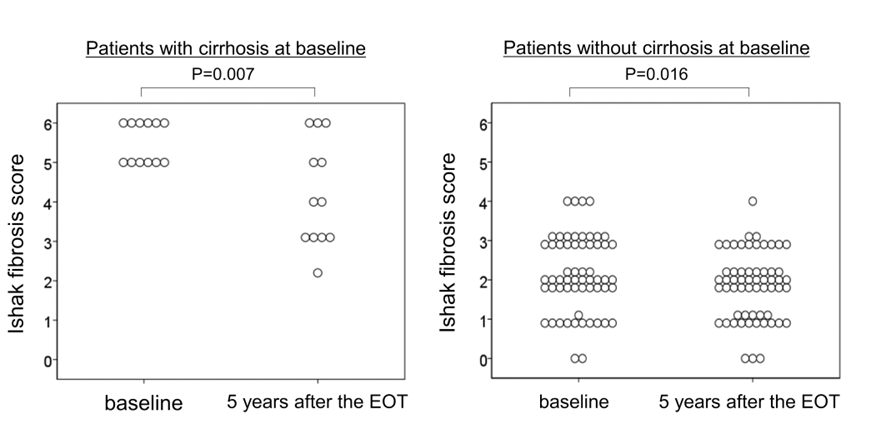
**

**Supplementary Figure S4.** Change in Ishak fibrosis score in patients with and without cirrhosis (Ishak fibrosis score ≥ 5) at baseline.

There were 12 and 59 patients with and without advanced fibrosis, respectively. The Ishak fibrosis score decreased significantly in patients with and without cirrhosis.


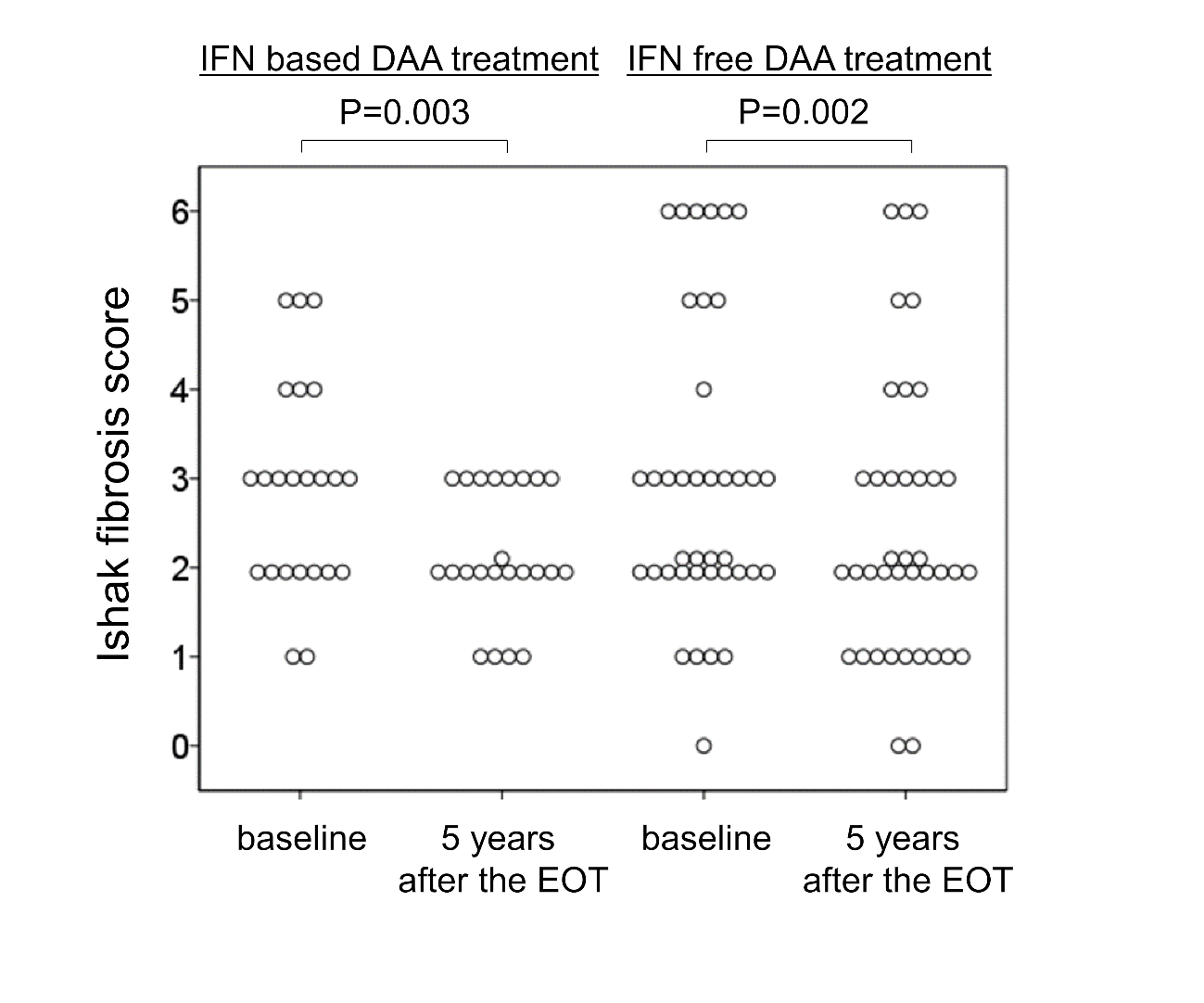


**Supplementary Figure S5.** Change in Ishak fibrosis score in patients with hepatitis C virus genotype 1 at baseline and 5 years after the end of treatment.

There were 23 and 39 patients treated with interferon (IFN)-based and IFN-free direct-acting antiviral treatment, respectively. The Ishak fibrosis score decreased significantly in both groups.


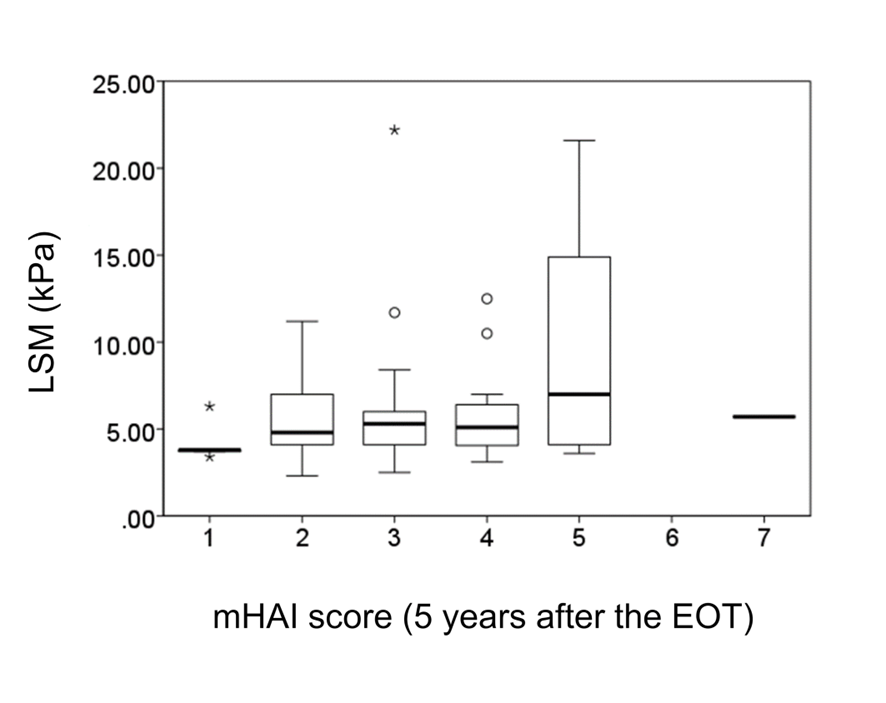


**Supplementary Figure S6.** The relationship between modified histological activity index (mHAI) score and liver stiffness measurements (LSM) at 5 years after the end of treatment.

There was no tendency for LSM to increase with increasing mHAI score (P=0.23).

The horizontal line through each box represents the median, and each box represents data from the 25th to the 75th percentile. The separate asterisks and circles represent outliers.

| **Table S1. P values for continuous hyaluronic acid and FIB-4 index.** | | | | | | | | | | | | |
| --- | --- | --- | --- | --- | --- | --- | --- | --- | --- | --- | --- | --- |
|  | P values for hyaluronic acid | | | | | | P values for FIB-4 index | | | | | |
|  | vs.  SVR24 | vs.  1 year | vs.  2 years | vs.  3 years | vs.  4 years | vs.  5 years | vs.  SVR24 | vs.  1 year | vs.  2 years | vs.  3 years | vs.  4 years | vs.  5 years |
| Baseline | < 0.001 | < 0.001 | < 0.001 | < 0.001 | < 0.001 | < 0.001 | < 0.001 | < 0.001 | < 0.001 | < 0.001 | < 0.001 | < 0.001 |
| SVR24 | - | 0.663 | 0.04 | 0.013 | 1.000 | 1.000 | - | 0.029 | 0.254 | 0.001 | < 0.001 | < 0.001 |
| 1 year | - | - | 1.000 | 1.000 | 1.000 | 1.000 | - | - | 1.000 | 0.086 | 0.011 | 0.013 |
| 2 years | - | - | - | 1.000 | 1.000 | 1.000 | - | - | - | 1.000 | 0.143 | 0.048 |
| 3 years | - | - | - | - | 1.000 | 1.000 | - | - | - | - | 1.000 | 0.823 |
| 4 years | - | - | - | - | - | 1.000 | - | - | - | - | - | 1.000 |
| FIB-4: Fibrosis 4. SVR: sustained virological response. | | | | | | | | | | | | |

| **Table S2.**  **Baseline characteristics of IFN-based DAA treatment group and IFN-free DAA treatment group.** | | | |
| --- | --- | --- | --- |
| Factor | IFN-based DAA treatment group (n=23) | IFN-free DAA treatment group (n=48) | P value |
| Age, years (IQR) | 64 (59.5-68.0) | 65 (59.5-71.0) | 0.46 |
| Sex (male/female) | 6/17 | 17/31 | 0.59 |
| Body mass index, kg/m^2^ (IQR) | 22.3 (21.3-24.7) | 22.7 (21.5-25.3) | 0.87 |
| Habitual alcohol intake 20-60 g/day, n (%) | 4 (17.4) | 1 (2.1) | 1.00 |
| History of diabetes mellitus treatment, n (%) | 10 (43.5) | 2 (4.2) | 0.31 |
| Treatment-naïve of HCV, n (%) | 16 (69.6) | 29 (60.4) | 0.60 |
| Past HCC treatment, n (%) | 1 (4.3) | 0 (0) | 0.32 |
| HbA1c, % (IQR) | 5.6 (5.4-6.1) | 5.7 (5.4-6.0) | 0.51 |
| HCV genotype (1/2) | 23/0 | 39/9 | 0.03 |
| HCV RNA, log_10_ IU/mL (IQR) | 6.4 (5.7-6.9) | 6.2 (5.5-6.6) | 0.13 |
| Aspartate aminotransferase, U/L (IQR) | 49 (32.0-68.5) | 35 (27.0-63.5) | 0.26 |
| Alanine aminotransferase, U/L (IQR) | 43 (32.0-73.0) | 37 (23.5-65.0) | 0.27 |
| Albumin, g/dL (IQR) | 4.3 (3.9-4.4) | 4.1 (3.9-4.3) | 0.20 |
| Platelet count, ×10^3^/μL (IQR) | 126.0 (106.5-156.0) | 157.5 (113.0-189.0) | 0.08 |
| α-fetoprotein, ng/mL (IQR) | 7 (5-13) | 6 (5-10) | 0.53 |
| Hyaluronic acid, ng/mL (IQR) | 129 (68.5-188.0) | 94 (39.0-221.5) | 0.49 |
| Type4 collagen 7S, ng/mL (IQR) | 5.7 (4.8-6.9) | 5.2 (4.0-7.1) | 0.37 |
| M2BPGi, unit (IQR) | 2.2 (1.2-2.8) | 1.7 (1.1-3.0) | 0.73 |
| Autotaxin, mg/mL (IQR) | 1.7 (1.0-1.9) | 1.4 (1.1-2.1) | 0.96 |
| FIB-4 index (IQR) | 3.74 (2.83-4.28) | 2.58 (1.89-4.19) | 0.08 |
| FIB-4: Fibrosis 4. HbA1c: glycated hemoglobin A1c. HCC: hepatocellular carcinoma. HCV RNA: hepatitis C virus ribonucleic acid. IFN: interferon, IQR: interquartile range. M2BPGi: Mac-2 binding protein glycosylation isomer. | | | |

| **Table S3.**  **Baseline characteristics of patients with and without advanced fibrosis (Ishak fibrosis score ≥ 4).** | | | |
| --- | --- | --- | --- |
| Factor | Patients with  advanced fibrosis (n=16) | Patients without  advanced fibrosis (n=55) | P value |
| Age, years (IQR) | 66 (62.5-69.5) | 64 (58.5-71.0) | 0.86 |
| Sex (male/female) | 5/11 | 18/37 | 1.00 |
| Body mass index, kg/m^2^ (IQR) | 23.6 (21.3-25.6) | 22.4 (21.5-24.9) | 0.40 |
| Habitual alcohol intake 20-60 g/day, n (%) | 0 (0) | 5 (9.1) | 0.58 |
| History of diabetes mellitus treatment, n (%) | 3 (18.8) | 9 (16.4) | 1.00 |
| Treatment-naïve of HCV (%) | 8 (50.0) | 37 (67.3) | 0.25 |
| Past HCC treatment (%) | 1 (6.3) | 0 (0) | 0.23 |
| HbA1c, % (IQR) | 5.8 (5.4-6.1) | 5.7 (5.4-6.0) | 0.56 |
| HCV genotype (1/2) | 16/0 | 46/9 | 0.19 |
| HCV RNA, log_10_ IU/mL (IQR) | 6.2 (5.6-6.7) | 6.3 (5.6-6.7) | 0.87 |
| Aspartate aminotransferase, U/L (IQR) | 61.5 (42.0-108.5) | 34.0 (27.0-52.0) | <0.01 |
| Alanine aminotransferase, U/L (IQR) | 54.5 (39.0-107.5) | 35.0 (23.0-57.0) | 0.02 |
| Albumin, g/dL (IQR) | 3.9 (3.6-4.2) | 4.2 (4.1-4.3) | <0.01 |
| Platelet count, ×10^3^/μL (IQR) | 117.0 (88.5-151.0) | 158.0 (115.0-186.0) | <0.01 |
| α-fetoprotein, ng/mL (IQR) | 15 (6.5-123.0) | 5 (5.0-7.0) | <0.01 |
| Hyaluronic acid, ng/mL (IQR) | 200 (75.5-393.5) | 92 (39.0-174.5) | 0.02 |
| Type4 collagen 7S, ng/mL (IQR) | 7.1 (6.6-10.5) | 5.0 (4.1-5.9) | <0.01 |
| M2BPGi, unit (IQR) | 3.9 (2.0-9.9) | 1.6 (1.0-2.2) | <0.01 |
| Autotaxin, mg/mL (IQR) | 2.3 (1.7-2.8) | 1.4 (0.9-1.8) | <0.01 |
| FIB-4 index (IQR) | 4.70 (3.51-7.62) | 2.50 (1.85-3.73) | <0.01 |
| FIB-4: Fibrosis 4. HbA1c: glycated hemoglobin A1c. HCC: hepatocellular carcinoma. HCV RNA: hepatitis C virus ribonucleic acid. IQR: interquartile range. M2BPGi: Mac-2 binding protein glycosylation isomer. | | | |

| **Table S4. P values for comparison between each noninvasive test at baseline.** | | | | | | | | |
| --- | --- | --- | --- | --- | --- | --- | --- | --- |
|  | Advanced fibrosis (Ishak fibrosis score ≥ 4) | | | | Cirrhosis (Ishak fibrosis score ≥ 5) | | | |
|  | vs.  4COL7S | vs.  M2BPGi | vs.  ATX | vs.  FIB-4 index | vs.  4COL7S | vs.  M2BPGi | vs.  ATX | vs.  FIB-4 index |
| HA | 0.074 | 0.119 | 0.090 | 0.027 | 0.229 | 0.392 | 0.342 | 0.359 |
| 4COL7S | - | 0.599 | 0.964 | 0.873 | - | 0.611 | 0.912 | 0.726 |
| M2BPGi |  |  | 0.613 | 0.502 |  |  | 0.600 | 0.860 |
| ATX | - | - |  | 0.827 | - | - |  | 0.772 |
| 4COL7S: Type4 collagen 7S. ATX: Autotaxin. FIB-4: Fibrosis 4. HA: Hyaluronic acid. LSM: liver stiffness measurement. M2BPGi: Mac-2 binding protein glycosylation isomer. | | | | | | | | |

| **Table S5. P values for comparison between each noninvasive test at five years after the EOT.** | | | | | | | | | | |
| --- | --- | --- | --- | --- | --- | --- | --- | --- | --- | --- |
|  | Advanced fibrosis (Ishak fibrosis score ≥ 4) | | | | | Cirrhosis (Ishak fibrosis score ≥ 5) | | | | |
|  | vs.  4COL7S | vs.  M2BPGi | vs.  ATX | vs.  FIB-4 index | vs.  LSM | vs.  4COL7S | vs.  M2BPGi | vs.  ATX | vs.  FIB-4 index | vs.  LSM |
| HA | 0.252 | 0.222 | 0.801 | 0.803 | 0.037 | 0.408 | 0.700 | 0.821 | 0.633 | 0.350 |
| 4COL7S | - | 0.002 | 0.106 | 0.075 | < 0.001 | - | 0.013 | 0.304 | 0.447 | 0.006 |
| M2BPGi | - | - | 0.081 | 0.133 | 0.071 | - | - | 0.093 | 0.295 | 0.215 |
| ATX | - | - | - | 0.942 | 0.032 | - | - | - | 0.885 | 0.089 |
| FIB-4 index | - | - | - | - | 0.007 | - | - | - | - | 0.074 |
| 4COL7S: Type4 collagen 7S. ATX: Autotaxin. FIB-4: Fibrosis 4. HA: Hyaluronic acid. LSM: liver stiffness measurement. M2BPGi: Mac-2 binding protein glycosylation isomer. | | | | | | | | | | |

| **Table S6. Predictive performance of non-invasive tests for cirrhosis at baseline.** | | | | | | | |
| --- | --- | --- | --- | --- | --- | --- | --- |
| Factor | AUROC | Cut-off | Sensitivity | Specificity | PPV | NPV | Accuracy |
| Hyaluronic acid | 0.809 | 221.0 | 0.667 | 0.864 | 0.500 | 0.927 | 0.831 |
| Type4 collagen 7S | 0.870 | 6.40 | 0.917 | 0.780 | 0.458 | 0.979 | 0.803 |
| M2BPGi | 0.847 | 2.28 | 0.833 | 0.763 | 0.417 | 0.957 | 0.775 |
| Autotaxin | 0.865 | 2.12 | 0.750 | 0.898 | 0.600 | 0.946 | 0.873 |
| FIB-4 index | 0.854 | 3.78 | 0.833 | 0.763 | 0.417 | 0.957 | 0.775 |
| AUROC: Area Under the Receiver Operating Characteristic. FIB-4: Fibrosis 4. M2BPGi: Mac-2 binding protein glycosylation isomer. NPV: negative predictive value. PPV: positive predictive value. | | | | | | | |

| **Table S7.**  **Predictive performance of non-invasive tests for cirrhosis at five years after the EOT.** | | | | | | | |
| --- | --- | --- | --- | --- | --- | --- | --- |
| Factor | AUROC | Cut-off | Sensitivity | Specificity | PPV | NPV | Accuracy |
| Hyaluronic acid | 0.836 | 90.0 | 0.800 | 0.864 | 0.308 | 0.983 | 0.859 |
| Type4 collagen 7S | 0.698 | 2.95 | 0.800 | 0.591 | 0.129 | 0.975 | 0.606 |
| M2BPGi | 0.897 | 2.03 | 0.800 | 0.970 | 0.667 | 0.985 | 0.958 |
| Autotaxin | 0.805 | 1.42 | 0.800 | 0.833 | 0.267 | 0.982 | 0.831 |
| FIB-4 index | 0.777 | 1.95 | 1.000 | 0.515 | 0.135 | 1.000 | 0.549 |
| LSM | 0.965 | 6.75 | 1.000 | 0.825 | 0.312 | 1.000 | 0.838 |
| AUROC: Area Under the Receiver Operating Characteristic. EOT: end of treatment. FIB-4: Fibrosis 4.  LSM: liver stiffness measurement. M2BPGi: Mac-2 binding protein glycosylation isomer.  NPV: negative predictive value. PPV: positive predictive value. | | | | | | | |

| **Table. S8**  **Clinical characteristics of patients with histological fibrosis progression and patients without histological fibrosis progression at baseline.** | | | |
| --- | --- | --- | --- |
| Factor | Patients with histological fibrosis progression (n=12) | Patients without histological fibrosis progression (n=59) | P value |
| Age, years (IQR) | 65 (61.0-70.5) | 65 (58.5-71.5) | 0.85 |
| Sex (male/female) | 4/8 | 19/40 | 1.00 |
| Body mass index, kg/m^2^ (IQR) | 25.3 (23.3-25.5) | 22.3 (21.2-23.7) | 0.01 |
| Habitual alcohol intake 20-60 g/day | 3 (25.0) | 2 (3.4) | 0.03 |
| History of diabetes mellitus treatment, n (%) | 3 (25.0) | 9 (15.3) | 0.41 |
| HbA1c, % (IQR) | 6.0 (5.6-6.5) | 5.7 (5.4-6.0) | 0.12 |
| Aspartate aminotransferase, U/L (IQR) | 25.5 (16.5-27.5) | 46.0 (32.5-69.0) | <0.01 |
| Alanine aminotransferase, U/L (IQR) | 21.0 (16.5-30.5) | 41.0 (31.5-75.0) | <0.01 |
| Platelet count, ×10^3^/μL (IQR) | 157.5 (118.0-171.0) | 150.0 (108.5-182.5) | 0.63 |
| α-fetoprotein, ng/mL (IQR) | 5 (5-5) | 6 (5-14) | 0.02 |
| Hyaluronic acid, ng/mL (IQR) | 39.0 (31.5-89.5) | 129.0 (50.0-221.0) | 0.02 |
| Type4 collagen 7S, ng/mL (IQR) | 2.9 (2.7-4.6) | 5.7 (4.8-7.4) | <0.01 |
| M2BPGi, unit (IQR) | 1.0 (0.7-1.6) | 1.8 (1.2-3.6) | <0.01 |
| Autotaxin, mg/mL (IQR) | 1.2 (0.8-1.6) | 1.5 (1.1-2.1) | 0.05 |
| FIB-4 index (IQR) | 2.09 (1.52-2.84) | 3.37 (2.13-4.38) | 0.01 |
| Quantitative assessment of hepatic steatosis, % (IQR) | 1.20 (0.68-2.45) | 1.88 (0.90-3.94) | 0.33 |
| mHAI score ≥ 4, n (%) | 3 (25.0) | 47 (79.7) | 1.00 |
| FIB-4: Fibrosis 4. HbA1c: glycated hemoglobin A1c. HCC: hepatocellular carcinoma. HCV RNA: hepatitis C virus ribonucleic acid. IFN: interferon, IQR: interquartile range. M2BPGi: Mac-2 binding protein glycosylation isomer. | | | |

| **Table. S9**  **Clinical characteristics of patients with histological fibrosis progression and patients without histological fibrosis progression at five years after the EOT.** | | | |
| --- | --- | --- | --- |
| Factor | Patients with histological fibrosis progression (n=12) | Patients without histological fibrosis progression (n=59) | P value |
| Age, years (IQR) | 70.5 (63.5-76.5) | 70.0 (67.0-76.0) | 0.89 |
| Sex (male/female) | 4/8 | 19/40 | 1.00 |
| Body mass index, kg/m^2^ (IQR) | 25.4 (23.6-26.5) | 22.5 (20.2-24.3) | 0.02 |
| Habitual alcohol intake 20-60 g/day | 3 (25.0) | 2 (3.4) | 0.03 |
| History of diabetes mellitus treatment, n (%) | 3 (25.0) | 16 (27.1) | 1.00 |
| Aspartate aminotransferase, U/L (IQR) | 20 (16-23) | 22 (19-24) | 0.18 |
| Alanine aminotransferase, U/L (IQR) | 16 (13-20) | 15 (12-22) | 0.92 |
| Platelet count, ×10^3^/μL (IQR) | 180.0 (148.3-189.8) | 175.0 (150.0-212.0) | 0.89 |
| α-fetoprotein, ng/mL (IQR) | 2 (2.0-3.5) | 3 (2.0-4.0) | 0.19 |
| Hyaluronic acid, ng/mL (IQR) | 63.5 (31.0-110.0) | 43.0 (27.0-75.0) | 0.16 |
| Type4 collagen 7S, ng/mL (IQR) | 2.4 (2.1-3.0) | 2.9 (2.5-4.1) | 0.04 |
| M2BPGi, unit (IQR) | 0.9 (0.7-1.2) | 0.9 (0.7-1.2) | 0.97 |
| Autotaxin, mg/mL (IQR) | 1.0 (0.8-1.2) | 1.2 (0.9-1.4) | 0.13 |
| FIB-4 index (IQR) | 1.93 (1.55-2.53) | 2.00 (1.64-2.40) | 0.72 |
| Quantitative assessment of hepatic steatosis, % (IQR) | 2.57 (1.46-7.80) | 2.24 (1.44-3.74) | 0.55 |
| mHAI score ≥ 4, n (%) | 6 (50.0) | 20 (33.9) | 0.34 |
| FIB-4: Fibrosis 4. HCC: hepatocellular carcinoma. HCV RNA: hepatitis C virus ribonucleic acid.  IFN: interferon, IQR: interquartile range. M2BPGi: Mac-2 binding protein glycosylation isomer. | | | |

| **Table. S10**  **Characteristics of patients with and without high LSM at 5years after the EOT.** | | | |
| --- | --- | --- | --- |
| Factor | Patients with high LSM (≥ 6.75 kPa) (n=16) | Patients without high LSM (<6.74 kPa) (n=55) | P value |
| Age, years (IQR) | 73.5 (66.5-77.0) | 69.0 (64.0-75.0) | 0.26 |
| Sex (male/female) | 5/11 | 18/37 | 1.00 |
| Body mass index, kg/m^2^ (IQR) | 23.9 (20.4-25.6) | 22.5 (20.5-24.3) | 0.34 |
| Habitual alcohol intake 20-60 g/day | 1 (6.3) | 4 (7.3) | 1.00 |
| History of diabetes mellitus treatment, n (%) | 6 (37.5) | 13 (23.6) | 0.34 |
| Aspartate aminotransferase, U/L (IQR) | 23 (21.0-24.5) | 21 (18.0-23.5) | 0.10 |
| Alanine aminotransferase, U/L (IQR) | 16.5 (14.0-23.5) | 15.0 (12.0-21.5) | 0.42 |
| Albumin, g/dL (IQR) | 4.2 (4.0-4.4) | 4.3 (4.1-4.4) | 0.47 |
| Platelet count, ×10^3^/μL (IQR) | 161.0 (137.5-184.0) | 184.0 (155.0-217.5) | 0.05 |
| α-fetoprotein, ng/mL (IQR) | 3 (2.0-3.5) | 3 (2.0-4.0) | 0.86 |
| Hyaluronic acid, ng/mL (IQR) | 77.5 (33.5-114.0) | 40.0 (27.0-67.0) | 0.04 |
| Type4 collagen 7S, ng/mL (IQR) | 3.1 (2.7-3.6) | 2.7 (2.4-3.9) | 0.24 |
| M2BPGi, unit (IQR) | 1.2 (0.9-2.0) | 0.8 (0.7-1.1) | 0.007 |
| Autotaxin, mg/mL (IQR) | 1.3 (1.0-1.6) | 1.1 (0.9-1.3) | 0.09 |
| FIB-4 index (IQR) | 2.29 (1.97-2.86) | 1.92 (1.52-2.32) | 0.02 |
| Quantitative assessment of hepatic steatosis, % (IQR) | 1.84 (1.44-3.60) | 2.36 (1.47-5.70) | 0.53 |
| Controlled attenuation parameter, dB/m (IQR) | 245.5 (214.0-274.5) | 230.5 (193.5-262.0) | 0.25 |
| FIB-4: Fibrosis 4. HCC: hepatocellular carcinoma. HCV RNA: hepatitis C virus ribonucleic acid.  IFN: interferon, IQR: interquartile range. M2BPGi: Mac-2 binding protein glycosylation isomer. | | | |
